# Experimental Analysis on the Influence and Optimization of μ-RUM Parameters in Machining Alumina Bioceramic

**DOI:** 10.3390/ma12040616

**Published:** 2019-02-18

**Authors:** Basem M. A. Abdo, Saqib Anwar, Abdulaziz M. El-Tamimi, Emad Abouel Nasr

**Affiliations:** 1 Industrial Engineering Department, College of Engineering, King Saud University, P.O. Box 800, Riyadh 11421, Saudi Arabia; sanwar@ksu.edu.sa (S.A.); atamimi@ksu.edu.sa (A.M.E.-T.); eabdelghany@ksu.edu.sa (E.A.N.); 2 Advanced Manufacturing Institute, King Saud University, Riyadh 11421, Saudi Arabia; 3 Faculty of Engineering, Mechanical Engineering Department, Helwan University, Cairo 11732, Egypt

**Keywords:** rotary ultrasonic machining, microchannels, alumina, surface roughness, edge chipping, optimization

## Abstract

Fabrication of precise micro-features in bioceramic materials is still a challenging task. This is because of the inherent properties of bioceramics, such as low fracture toughness, high hardness, and brittleness. This paper places an emphasis on investigating the multi-objective optimization of fabrication of microchannels in alumina (Al_2_O_3_) bioceramics by using rotary ultrasonic machining (RUM). The influence of five major input parameters, namely vibration frequency, vibration amplitude, spindle speed, depth of cut, and feed rate on the surface quality, edge chipping, and dimensional accuracy of the milled microchannels was analyzed. Surface morphology and microstructure of the machined microchannels were also evaluated and analyzed. Unlike in previous studies, the effect of vibration frequency on the surface morphology and roughness is discussed in detail. A set of designed experiments based on central composite design (CCD) method was carried out. Main effect plots and surface plots were analyzed to detect the significance of RUM input parameters on the outputs. Later, a multi-objective genetic algorithm (MOGA) was employed to determine the optimal parametric conditions for minimizing the surface roughness, edge chipping, and dimensional errors of the machined microchannels. The optimized values of the surface roughness (Ra and Rt), side edge chipping (SEC), bed edge chipping (BEC), depth error (DE), and width error (WE) achieved through the multi-objective optimization were 0.27 μm, 2.7 μm, 8.7 μm, 8 μm, 5%, and 5.2%, respectively.

## 1. Introduction

Bioceramics such as alumina, zirconia, and silicon carbide possess outstanding physical, chemical, and thermal properties. Accordingly, they have wide applications in the evolution of micro-products in the medical field, such as microfluidic devices, labs on chips, micro-reactors, and micro-mixers [1]. However, due to their superior thermal, mechanical, and electrical properties, bioceramics are classified as difficult-to-process materials. Moreover, the high machining costs of bioceramics restrict their widespread applications. Consequently, there is a need to find alternative processes to machine these components with less cost and high quality. Microchannels are the most common micro-features required in microfluidic devices which are utilized for transportation and mixing of different fluids [2]. Alumina (Al_2_O_3_) is one of the most commonly used materials in microfluidic applications due to its high biocompatibility [3]. Machining of precise microchannels in alumina (Al_2_O_3_) is critical for its applications. However, the efficient machining of microchannels in Al_2_O_3_ material is still a challenging task due to its low fracture toughness and high hardness [4]. Excessive edge chipping, high tool wear, and high cutting forces result in high processing costs of machining Al_2_O_3_ by traditional processes such as diamond grinding [5]. Even non-conventional processes (for example, the laser ablation process) produce thermal damage, and poor surface roughness and accuracy [6,7]. Low material removal rate and poor dimensional accuracy have been the main drawbacks reported while machining alumina bioceramic using ultrasonic machining (USM) and abrasive machining [8]. Moreover, the inherent properties of Al_2_O_3_ bioceramic, such as its chemical inertness and poor electrical conductivity, impose a further limitation to its processing by chemical milling and electrical discharge machining.

**Figure 1:**
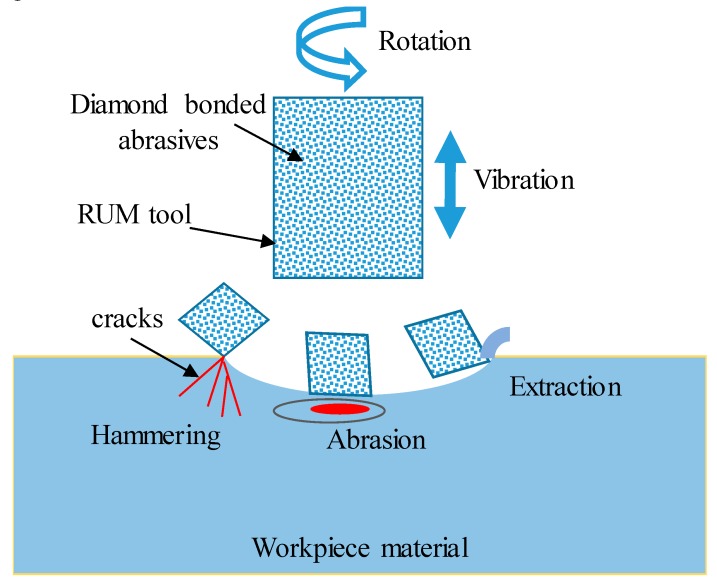
Mechanism of material removal of rotary ultrasonic machining (RUM).

Rotary ultrasonic machining (RUM) is one of the newer promising processing technologies used for overcoming the machining difficulties of bioceramics. It was developed as an alternative to USM. RUM is a hybrid non-conventional process which combines the mechanisms of traditional diamond grinding and static USM [9]. RUM is a purely mechanical process which can process both conductive and non-conductive materials without altering the mechanical and metallurgical characteristics of the machined material [9,10]. The main advantage of RUM is that it can produce parts with high accuracy and high surface quality [11]. The material removal mechanism of the RUM process can be categorized into three major modes, namely: (i) the hammering action of the tool due to the ultrasonic vibrations causing indentation and crushing; (ii) abrasion, resulting due to the rotational motion of the cutting tool; and (iii) extraction, due to the combined action of ultrasonic vibrations and rotation of the tool [9]. Figure 1 illustrates the three material removal mechanisms of the RUM process.

Recently, much research has elected to study the capability of RUM in drilling, milling, and grinding of brittle materials such as Al_2_O_3_, SiC, and glass. Most reported studies have focused on investigating the tool wear, edge chipping, material removal rate (MRR), surface roughness, and cutting forces. For example, an experimental study was performed by Gong et al. [12] to analyze the mechanism of RUM side milling of alumina ceramic. The results showed that the RUM has less tool wear than the grinding process. Popli and Gupta [13] applied RUM in drilling Al_2_O_3_ materials and found that the edge chipping can be reduced by increasing the support length to the workpiece. Liu et al. [14] investigated the tool wear and the cracking at the exit of the micro-holes (0.5 mm in diameter) drilled in Al_2_O_3_ using RUM. Response surface methodology was employed to minimize the exit chipping and tool wear. The optimization results showed that the tool wear and cracks in the hole were decreased to 16.891 μm and 25.375 μm, respectively, when a low feed rate (7 μm/s), an adequate amount of ultrasonic power (36%), and high cutting speed (1983 rpm) were applied. Singh and Singhal [15] proposed a quantified method for measuring the chipping size and MRR while drilling alumina ceramic using the RUM process. The results showed that the tool feed rate has the highest effect on both MRR and chipping size. Shen et al. [16] studied the wear behaviour of diamond grinding and ultrasonic assisted grinding process of alumina ceramic. The results revealed that the ultrasonic vibration improved the self sharpening property of the diamond grinding wheel and reduced the cutting force. RUM was also applied for the drilling of ceramic matrix composite materials C/SiC [17,18]. It was found that the drilling force and torque for the RUM process were decreased by 23% and 47.6%, respectively, when compared to the conventional drilling process [17]. The surface micro-cracks in ceramic materials machined using RUM were modelled by Shiliang et al. [19]. The results revealed that crack density increases with the increase in the cutting force while it decreases as fracture toughness increases. The effects of tool and RUM parameters on tool wear during the drilling of micro-holes (300 µm in diameter) in borosilicate glass were analyzed in the study documented in [20]. It was observed that by selecting a proper vibration frequency and using a tool of 100 μm wall thickness and diamond grain size of 30 μm, minimum tool wear could be achieved.

Furthermore, RUM has been effectively used to fabricate micro-channels in brittle materials such as borosilicate glass [21,22] and BK7 glass [23]. For example, Kumar and Akashy [21] investigated the effects of tool spindle speed, feed rate, ultrasonic power, and grit size on tool wear, form accuracy, and MRR while fabricating microchannels (0.6 mm in width) in borosilicate glass using RUM. It was demonstrated that longitudinal and edge rounding wear are the major factors that affect the RUM process. Authors recommended a compensation of 0.092 μm for each 1 μm depth of cut for longitudinal wear to improve the machined depth accuracy. Cheema et al. [24] studied the effect of tool material, abrasive size, and step feed in the fabrication of microchannels (0.6 mm in width and 0.3 mm in depth) by using RUM on borosilicate glass. Results showed that a better form accuracy of the machined microchannels could be achieved by using a tungsten carbide tool and small diameter abrasives.

From studies reported in the literature it can be found that very few little research has been conducted on the micro-milling of alumina while no research has focused on the machining of microchannels in alumina using RUM. The majority of reported studies have been limited to drilling experiments where the effect of a single input parameter on the cutting force and tool wear was studied. Moreover, most previously reported studies have been limited to the study of the effect of only three input parameters, with very few responses studied. The effect of “frequency (F)” has never been considered throughout all investigations conducted for the RUM of alumina bioceramic. Furthermore, a broad range (5 μm to 50 μm) of the vibration amplitude (ultrasonic power) is selected in the current study as compared to the previously utilized range of 5 μm to 30 μm. The present study is targeted to explore the impact of five major RUM input parameters, including spindle speed (S), feed rate (FR), depth of cut (DOC), vibration frequency (F), and vibration amplitude (A) on the micromachining of alumina. The outputs, including the surface roughness (Ra and Rt), dimensional accuracy (namely depth error (DE) and width error (WE)), and edge chipping (including side exit chipping (SEC) and bed exit chipping (BEC)), are studied. Surface morphology of the machined microchannels and the wear mechanisms of the RUM micro tools are also analyzed using scanning electron microscopy (SEM). Central composite design (CCD) based on a factorial design is used to analyze the effect of the multiple input parameters on the selected outputs. Moreover, the multi-objective genetic algorithm (MOGA) method is applied to simultaneously optimize the Ra, Rt, SEC, and BEC while keeping the DE and WE of the milled microchannels less than 8%. 

## 2. Materials and Experimental Procedure

### 2.1. Experimental Set Up

**Figure 2:**
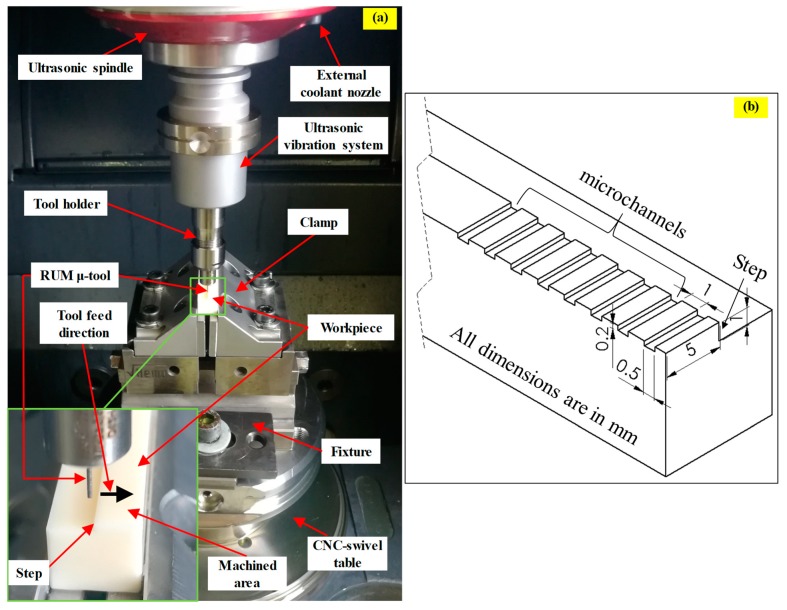
(**a**) RUM experimental set up; (**b**) 3D schematic of the microchannels.

The experiments in this study were conducted using Ultrasonic 20-Linear (DMG, Geretsried, Germany), which is a five-axis machine that can perform both traditional high-speed milling and RUM. The experimental setup is shown in Figure 2a. The key components of Ultrasonic 20-Linear consist of an ultrasonic spindle coupled to an ultrasonic transducer with a maximum rotation speed of 10,000 rpm, power supply, coolant system, an HSK32 ultrasonic actuator system, and an integrated NC-rotary table. An output peak power of 2000 watts is used to convert a 50 Hz AC into an ultrasonic frequency range of 18.5–48 kHz. The tool vibration amplitude ranges 5–80 µm and is controlled in terms of the percentage of the ultrasonic power (50% to 100%) via an ultrasonic generator.

### 2.2. Materials and Tools 

The RUM experiments were performed on alumina (Al_2_O_3_, 99.8%) bioceramic from CeramTec, Germany. Due to its brilliant biocompatibility, Al_2_O_3_ is graded as one of the most demanding materials in a wide range of medical applications [25]. All the samples used had dimensions 50 mm × 10 mm × 10 mm. The mechanical and thermal properties of the alumina samples are listed in Table 1.

**Figure 3:**
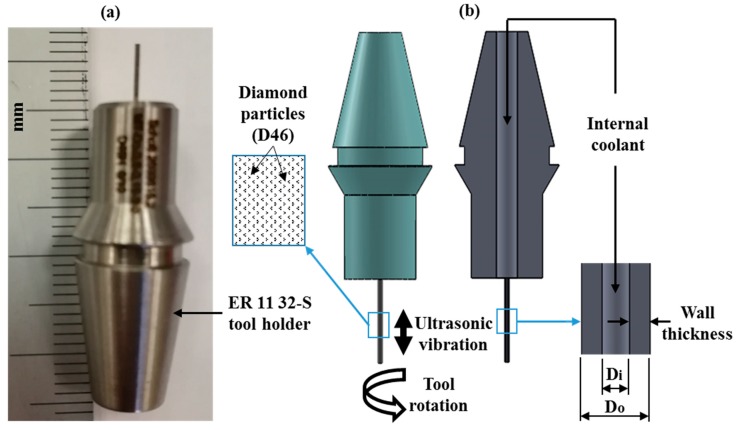
(**a**) Actual µ-RUM tool; (**b**) schematic of RUM tool.

The cross-sectional dimensions of the microchannel machined in this study are 0.5 mm (width) × 0.2 mm (depth), as shown in Figure 2b, which are commonly used microchannel dimensions in micro-fluidics applications [27,28,29]. The length of the microchannels was selected as 5 mm to get a more machined area in a single run for each channel. The workpiece material received from the CeramTec, Germany was pre-machined by using an 8 mm diameter RUM tool to create a step of 1 mm to ensure that the tool enters and leaves chamfer (0.5 mm 45°) free edges. Nickel bonded diamond µ-RUM tools made by Schott Company, Germany were used in the current study as presented in Figure 3a. The outer diameter (D_o_) and the inner diameter (D_i_) of the tools were 0.5 mm and 0.2 mm respectively (see Figure 3b). It should be noted that RUM tools up to 0.3 mm diameter are available, and can be used to machine micro channels/features of 300 µm or above.

### 2.3. Experimental Design and Selection of the Process Parameters

Central composite design was utilized to design the main experiments, aiming to identify the influence of RUM input parameters on the selected outputs. The CCD was formulated by using the statistical software “Minitab 17”. The CCD has the benefits of predicting a second-order behaviour of the response for a wider range of process parameters and to obtain quantitative information by conducting a small number of experiments [30]. A total of 32 runs was performed as per the half CCD experimental plan. Table 2 presents the RUM input parameters and their respective levels used in the experiments. The RUM parameters were selected based on the constraints imposed from the experimental setup, preliminary trials, and the literature reported on the micro-machining of brittle materials using RUM [20,21,31,32]. Other parameters such as coolant pressure (5 bar) and tool type were kept constant through all the experiments. 

### 2.4. Measurement Procedures

**Figure 4:**
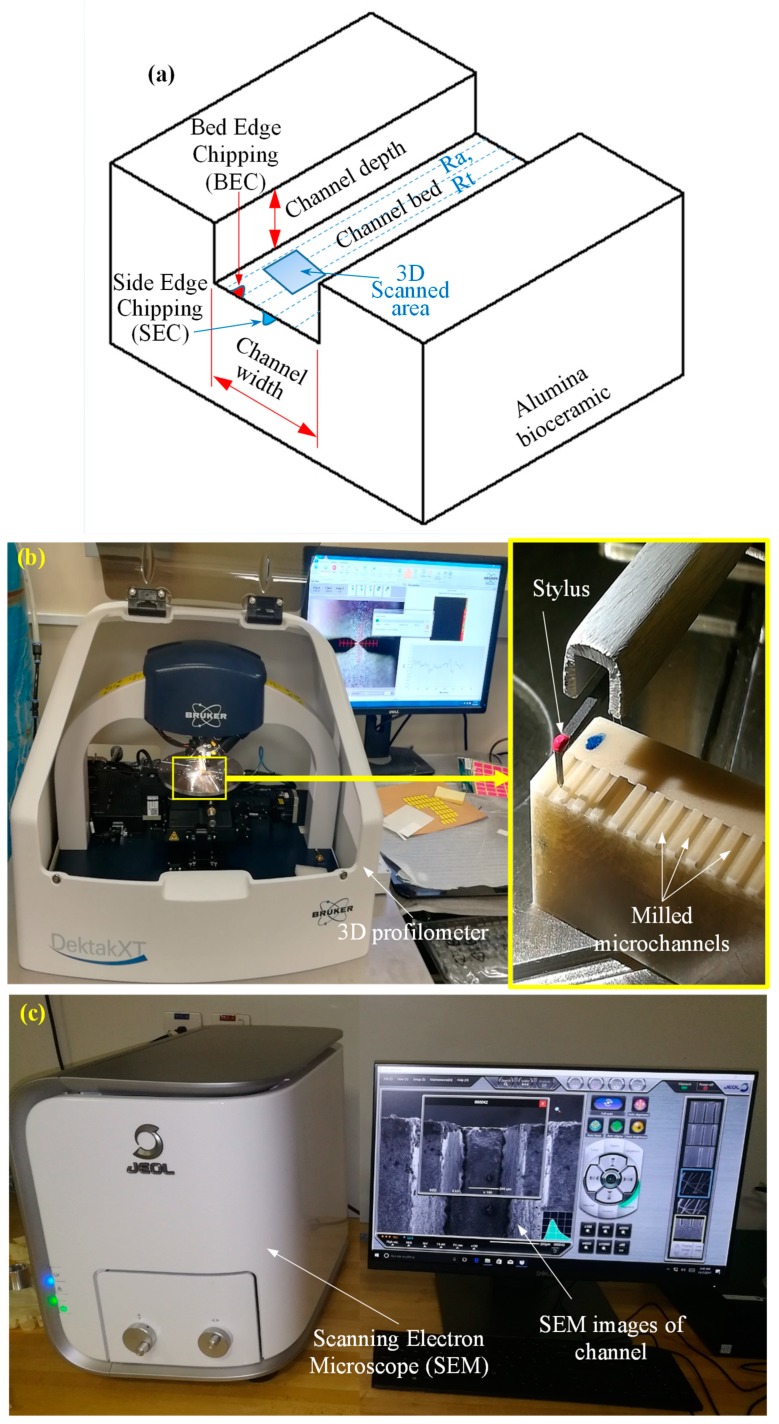
Outputs measurement procedure: (**a**) 3D schematic of the microchannel; (**b**) surface roughness measuring set up; and (**c**) surface morphology measuring set up.

The channels’ geometries, including channel width and channel depth (see Figure 4a), were measured using an optical microscopic. Later, the percentage of depth error and width error were used for further analyses. Surface roughness in terms of arithmetic mean roughness (Ra) and a maximum height of the roughness profile (Rt) were measured along each channel bed at five different locations using a 3D profilometer (DektakXT Stylus Profiler) from Bruker, USA. The average of these five readings was used for analysis. Figure 4b shows the setup of measuring the surface roughness. In order to enhance microchannel visibility during scanning electron miscroscopy (SEM) analysis, they were platinum-coated with a thickness of 10 μm using a JFC 1600 auto fine coater from JEOL Ltd. The surface morphology and the microstructure of the microchannels at the channels’ bed were observed using a tabletop SEM from Jeol Japan (Model JCM 6000Plus) as shown in Figure 4c. The exit edge chipping at the channel side, and at the channel bed (see Figure 4a) was also measured with the help of SEM. The RUM tools were observed under the optical microscope and SEM, before and after the machining, to understand the tool wear mechanisms. 

## 3. Results and Discussions

**Figure 5:**
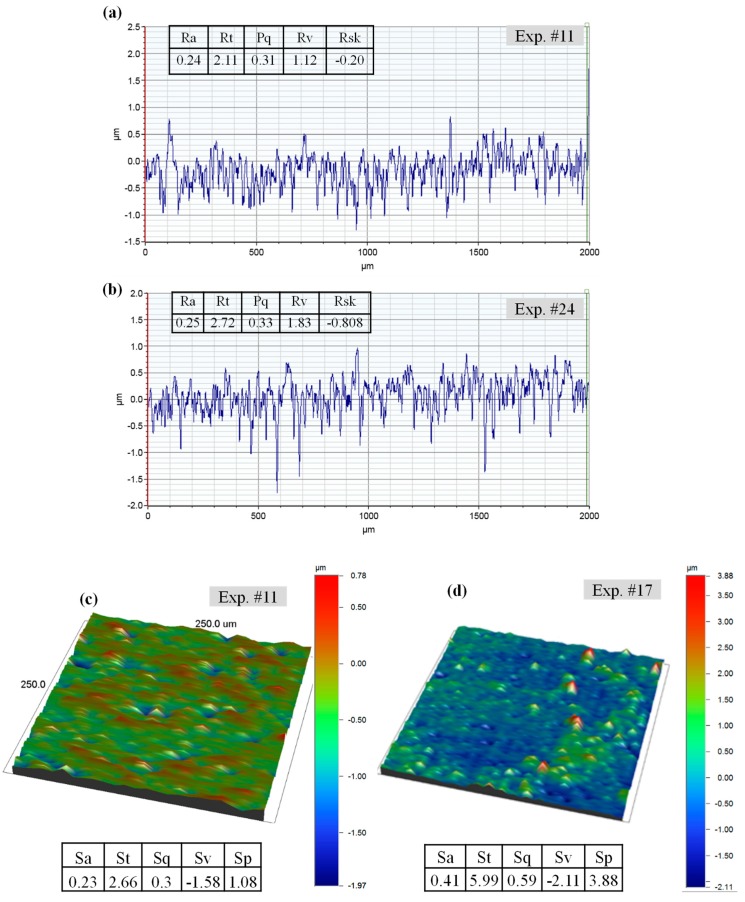
Example of typical scanned profiles milled at parameters of (**a**) 2D profile of Exp. #11, (**b**) 2D profile of Exp. #24, (**c**) 3D scanned area of Exp. #11, and (**d**) 3D scanned area of Exp. #24.

Table 3 lists the experimental results of 32 microchannels machined in alumina under several RUM parameters using the central composite design. Surface roughness is considered one of the important performance measures of the RUM process. The surface roughness (Ra and Rt) was measured at five random locations along the microchannel bed. The values of the Ra and Rt showed in Table 3 are the average of these five readings. Figure 5 shows examples of the typical scanned 2D and 3D profiles of the microchannels milled under various RUM parameters. It shows that the roughness profiles differ from smooth (Figure 5a,c) to rough (Figure 4b,d) and suggests that the RUM parameters have a considerable effect on the surface roughness and morphology of the milled microchannels. The percentage of the channel dimensional errors including depth error and width error were calculated by using Equations (1) and (2), respectively.

(1) $$\mathrm{Depth}\;\mathrm{error}\;\left(\mathrm{DE}\right)\;\%=|\frac{MD-DD}{DD}|\times 100$$

(2) $$\mathrm{Width}\;\mathrm{error}\;\left(\mathrm{WE}\right)\;\%=|\frac{MW-DW}{DW}|\times 100$$

MD is the measured depth after machining, DD is the desired depth, MW is the measured width after machining and DW is the desired width. The designed cross-sectional size of the milled microchannels was fixed at 500 µm in width and 200 µm in depth for all experiments. For example, in Experiment #1 (Exp. #1), the measured depth and the measured width were 211 µm and 545 µm, respectively. By applying Equations (1) and (2), the values of depth error and width error were calculated as 5.5% and 9%, respectively. The exit edge chipping at channel side and channel bed were measured at different locations (see Figure 4a). The average of the collected readings of SEC and BEC are reported in Table 3. 

**Figure 6:**
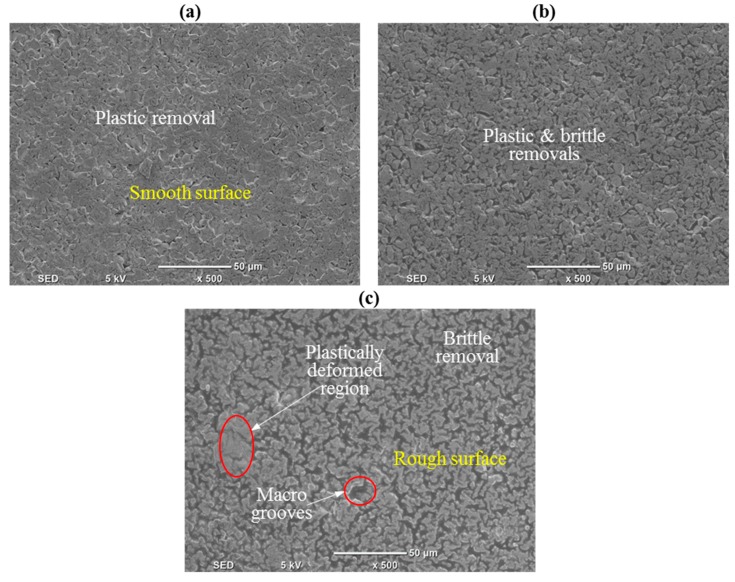
Scanning electron microscopy (SEM) micrograph of the machined channel for (**a**) Exp. #11, (**b**) Exp. #15, and (**c**) Exp. #32.

Figure 6a–c presents the SEM images of the processed surface as observed on the microchannel bed. These images correspond to Exp. #11 (Figure 6a), Exp. #15 (Figure 6b), and Exp. # 32 (Figure 6c). It can be observed from the SEM image shown in Figure 6a that plastic removal takes place owing to the increase in the contact time between the surface being machined and the diamond abrasives on the rotary tool occurring at higher levels of speed at 7000 rpm (higher grinding action) and low levels of feed rate and depth of cut. This results in the production of a smoother surface. On the other hand, a mixed mode of material removal (plastic removal and brittle fracture) with dominant plastic removal was found at moderate levels of speed, feed rate, and depth of cut (see Figure 6b). A machined surface with a dominant brittle fracture mode and deeper grooves is observed in Figure 6c, producing a rougher surface associated with higher levels of feed rate and depth of cut and lower levels of cutting speed. These results are in line with the findings of other authors machining brittle materials via RUM [15].

**Figure 7:**
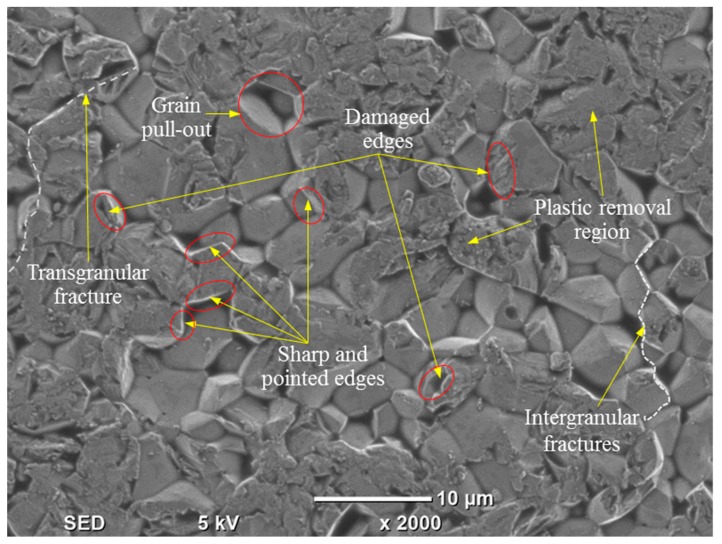
The microstructure of processed surface for Exp. #17.

To understand the microstructure developed after the RUM of alumina, a microchannel was observed in SEM under higher magnification. Figure 7 shows the microstructure of the microchannel milled in Exp. #17 at 2000× zoom. The microstructure shows a mix of the brittle fracture mode along with plastic removal of the alumina. In some regions pulled out grains can be observed leaving deep pits in the surface which later result in higher values of R_t_. The grains surrounding the pits exhibit both sharp and damaged boundaries [15]. Furthermore, both intergranular and transgranular fractures can be seen on the machined surface which results in increasing the surface roughness.

**Figure 8:**
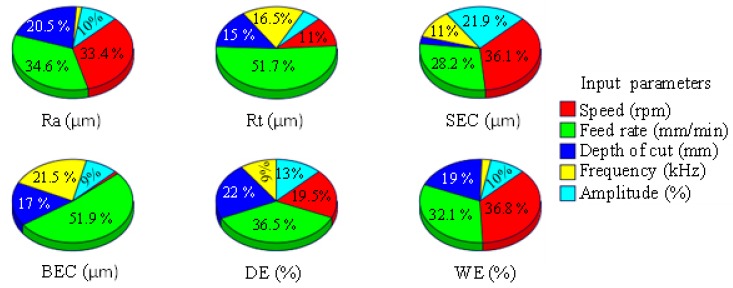
Percentage of the effect of RUM input parameters on the outputs. Legend: Ra, arithmetic mean roughness; Rt, maximum height of the roughness profile; SEC, side edge chipping; BEC, bed edge chipping; DE, depth error; WE, width error.

**Figure 9:**
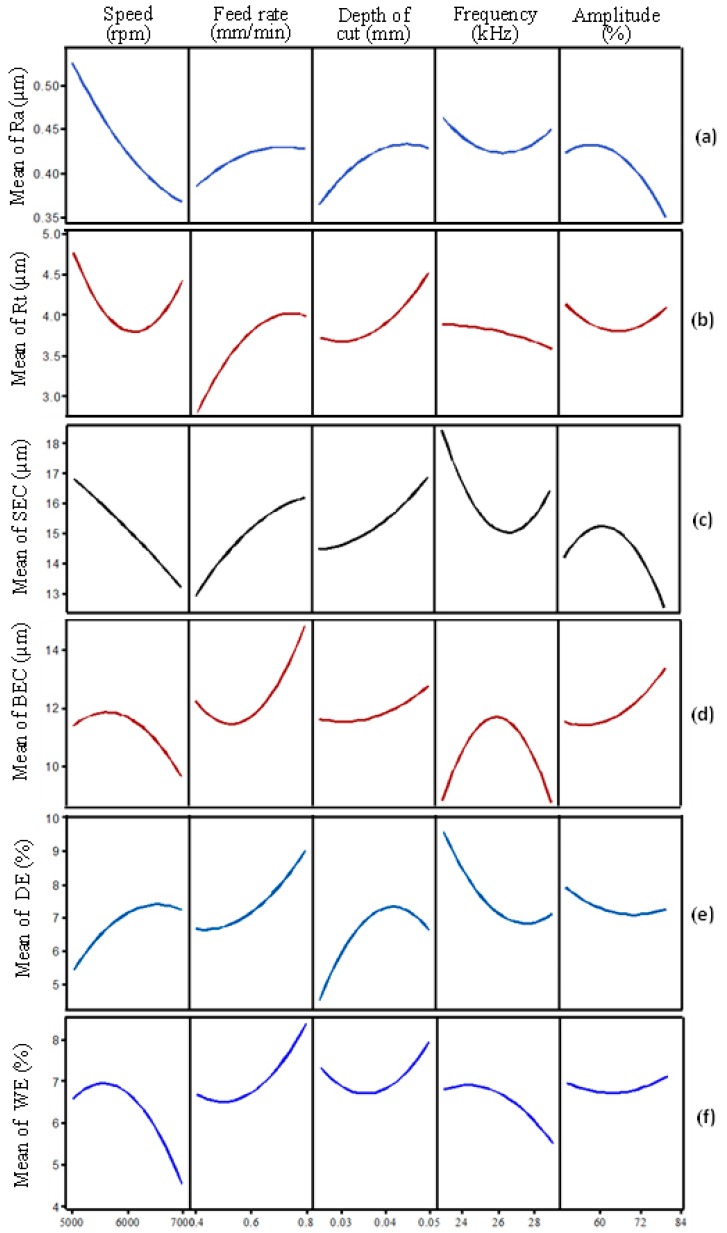
Main effect plots of RUM parameters on (**a**) Ra, (**b**) Rt, (**c**) SEC, (**d**) BEC, (**e**) DE, and (**f**) WE.

The relative significance of the RUM parameters on the outputs is illustrated in Figure 8. Among all input parameters, the feed rate was found to be the most influential, followed by the spindle speed and depth of cut. For any output, the feed rate contribution ranges between 30% to <52%. Ultrasonic parameters including frequency and amplitude show up to 22% contribution on surface roughness parameters (Ra and Rt), whereas regarding the edge chipping (SEC and BEC) and the depth error, the effect of the ultrasonic parameters reaches up to 33%. The trends of the outputs (Ra, Rt, SEC, BEC, DE, WE) with the variation of the RUM parameters are shown in the form of the main effect plots in Figure 9a–f.

### 3.1. Effects of µ-RUM Parameters on Surface Roughness

**Figure 10:**
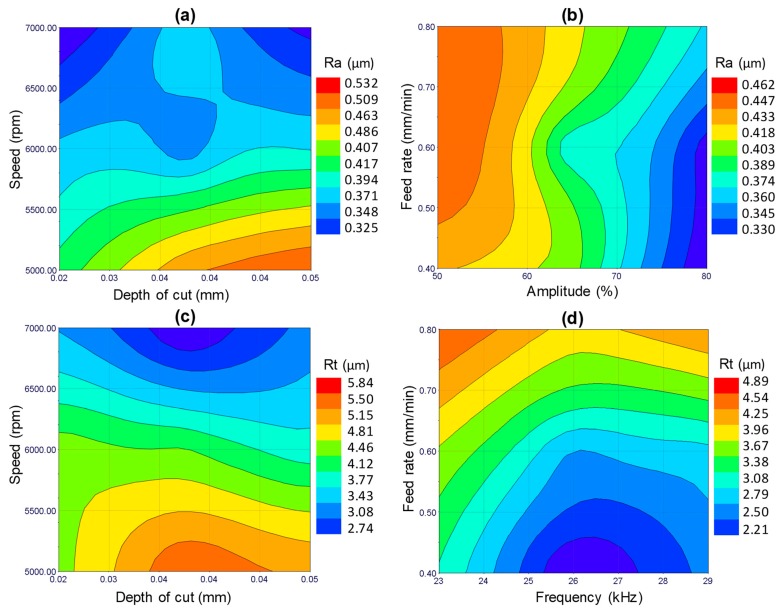
Response surface plots, i.e., effects of the indicated process parameters on (**a**) and (**b**) Ra, and (**c**) and (**d**) Rt_._

**Figure 11:**
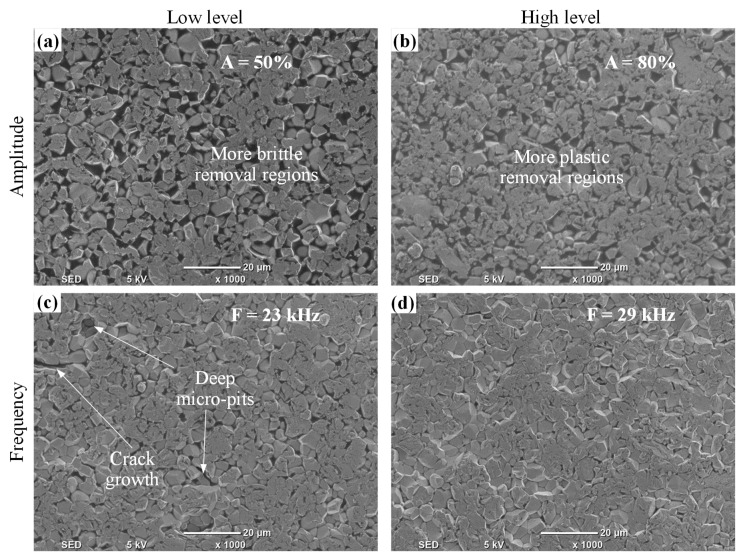
Effects of ultrasonic parameters on machined surface morphology at constant values (mid-ranges) on the remaining parameters: (**a**,**b**) effects of amplitudes and (**c**,**d**) effects of frequency.

**Figure 12:**
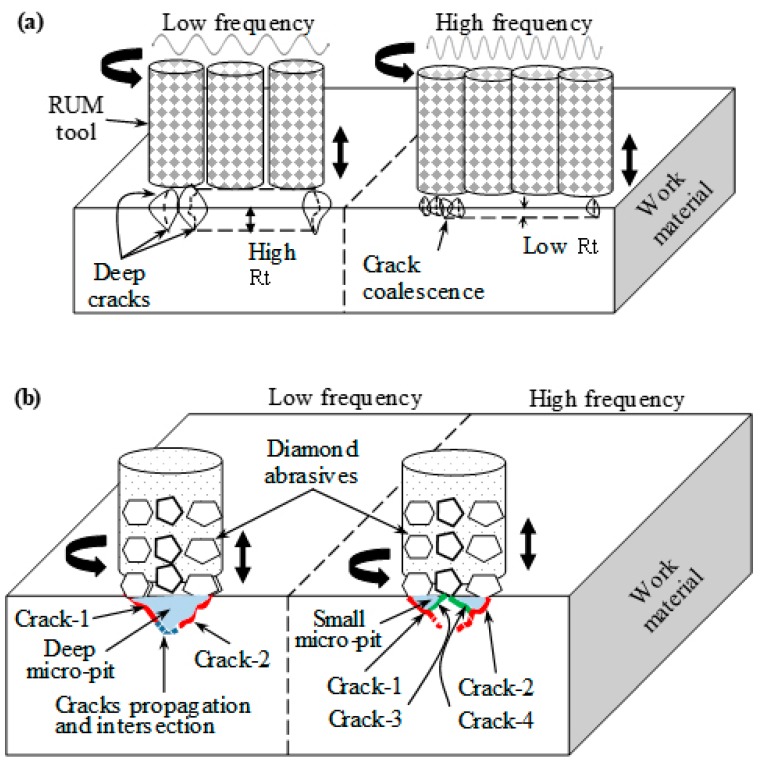
(**a**) Schematic illustration of the effect of vibration frequency on Rt; (**b**) crack propagation and coalescence.

The response surface plots for the surface roughness (Ra and Rt) are shown in Figure 10. For the sake of brevity, the surface plots for the outputs are presented only against the most significant RUM parameters shown earlier in Figure 8. For example, for Ra (see Figure 10a,b) the surface plots show the effects of the feed rate, spindle speed, and depth of cut and amplitude, as these are the most significant parameters regarding Ra. From Figure 9a, it is clear that the minimum value of Ra can be found at higher levels of spindle speed and lower values of depth of cut (see Figure 10a). Figure 9a also shows that an increase in the spindle speed from 5000 rpm to 7000 rpm causes a sharp decrease in Ra from 0.5 μm to 0.36 μm. This is because at higher cutting speed the contact time of the surface being machined and the diamond abrasives on the rotary tool increases, resulting in reducing the actual depth of cut per diamond abrasives and lowering the cutting force [33]. This results in more cutting action (plastic removal) and reducing the brittle fracture of the alumina, meaning a relatively smoother surface is produced. On the other hand, the increase in the feed rate causes an increase in Ra (see Figure 10b). This is because the high feed rate leads to more abrasive impact action and penetration depth on the machined surface that promotes more stress and consequently produces a rough surface [15]. The surface roughness (Ra and Rt) was observed to increase with increasing the depth of cut as shown in Figure 10a,c. This is again due to an increase in the cutting load per diamond abrasive [34] which results in more fracturing and deeper pit generation in the machined surface. The higher levels of amplitude and frequency result in minimum values of Ra and Rt respectively, as displayed in Figure 10b,d. When the vibration amplitude increases, the hammering action of the tool increases and consequently the pre-cracking in the material also increases, which makes the brittle material easier to be machined with more plastic removal, thus leading to enhanced Ra. The effect of increasing the amplitude on the surface morphology can be seen in Figure 11. At lower levels of the amplitude (see Figure 11a), more brittle removal is observed due to less hammering action while, more plastic removal can be seen in Figure 11b at the higher level of the amplitude. On the other hand, as the vibration frequency increases, the occurrence of deep micro-pits in the machined surface reduces. This can be seen by comparing Figure 11c,d where at a lower frequency, a higher number of micro-pits are produced (higher Rt) compared to smoothed morphology observed at a higher frequency. This increase in Rt due to the decrease in vibration frequency can also be observed in the 2D roughness profiles shown in Figure 5a,b. At higher frequency the crack density increases due to more frequent hammering of the tool and hence crack coalescence increases, decreasing crack propagation and leading to reduced Rt (see Figure 12a). At lower frequency, the individual randomly oriented cracks (see Crack-1 and Crack-2 in Figure 12b) have more time to propagate before these are intersected. On the contrary, at a higher frequency, the tool impacts the machined surface more often, resulting in more cracks (see Crack-3 and Crack-4 in Figure 12b), which intercept the previously generated cracks at lower depth under the fabricated surface and as a result decrease micro-pit size and Rt. 

### 3.2. Effects of µ-RUM Parameters on Edge Chipping

**Figure 13:**
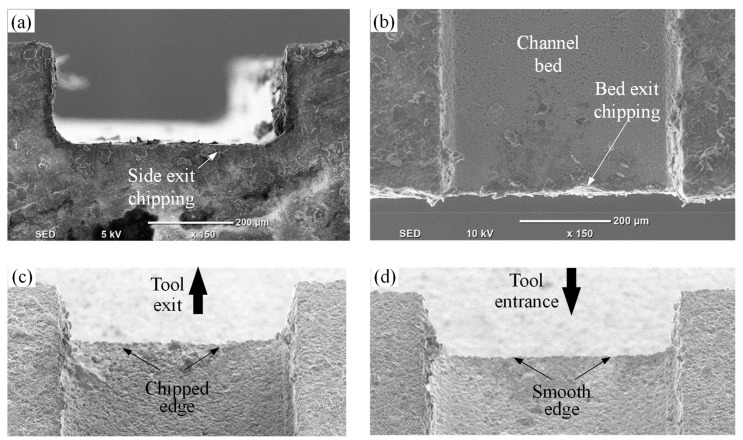
A comparison between entrance and exit edge chipping: (**a**) a typical micrograph of side edge chipping; (**b**) a typical micrograph of bed edge chipping; (**c**) edge chipping at the tool exit; and (**d**) edge chipping at the tool entrance edge.

**Figure 14:**
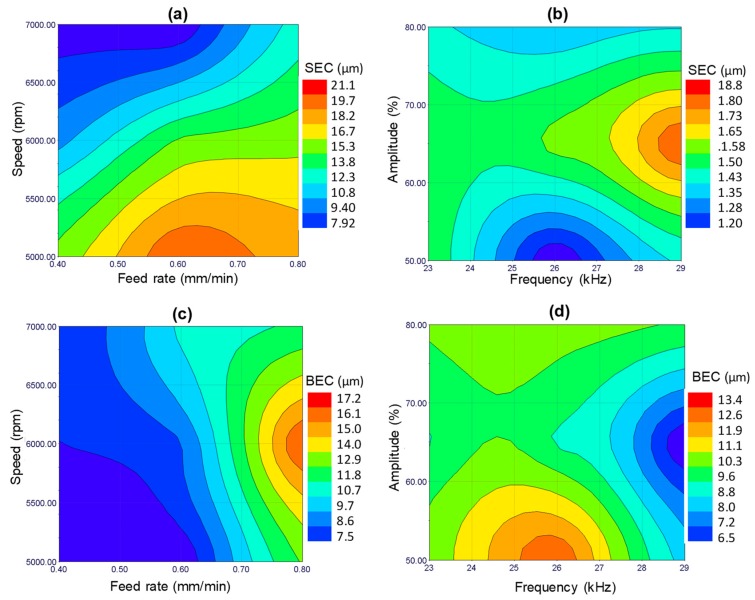
Response surface plots, i.e., effects of the indicated process parameters on (**a**) and (**b**) SEC, and (**c**) and (**d**) BEC.

In RUM, the damages from the machining mainly include edge chipping, which adversely affects the accuracy and reduces the strength of the product [35]. Therefore, to further access the quality of the machined microchannels, the edge chipping was measured on the channel bed and also along the tool exit plane (see Figure 4a). Figure 13a,b shows the typical micrograph of the bed exit edge chipping and the side exit edge chipping. It was found that BEC and SEC were always higher on the tool exit side as compared to the tool entrance side, as shown in Figure 13c,d. This is due to the tensile forces exerted on the exit edge by the RUM tool before exiting the channel; compressive forces are generated while entering the channel. All the presented BEC and SEC values correspond to the exit edge of the microchannels. Figure 14 shows the surface plots of the side exit chipping and bed edge chipping. It can be seen in Figure 14a,c that as the spindle speed increases and the feed rate decreases, the values of SEC and BEC reduce considerably. This happens because any change in input parameters which leads to an increase in the force between the tool and the workpiece means the edge chipping will increase [33,36]. Regarding the effect of ultrasonic parameters, no clear trend is observed, as shown in Figure 14b,d. In general, it can be said that the minimum values of exit edge chipping (BEC and SEC) are found at moderate to high levels of frequencies (25–28 kHz) and small to medium values of amplitudes (50–70%). This might be because of the complex material removal mechanism affected by the variation in the hammering action of the tool, crack length, crack density, and crack coalescence. 

### 3.3. Effects of µ-RUM Parameters on Dimensional Errors

**Figure 15:**
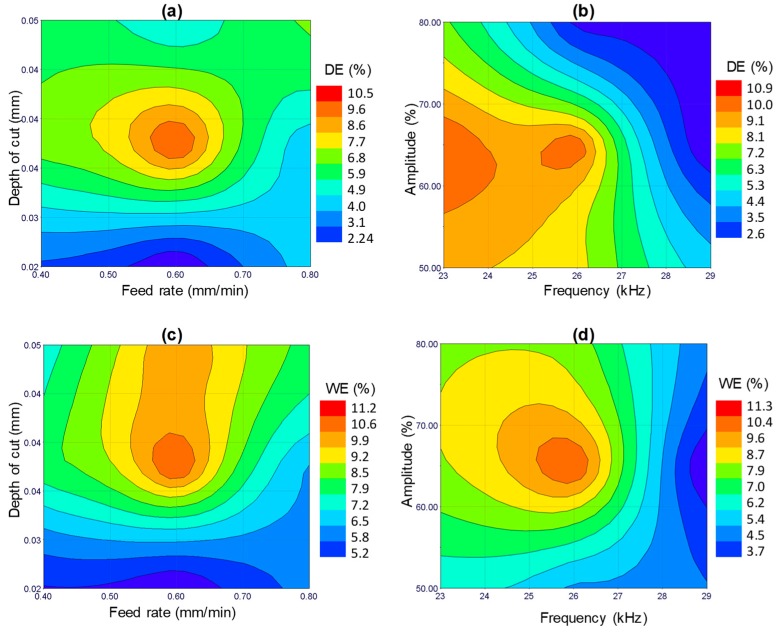
Response surface plots, i.e., effects of the indicated process parameters on (**a**) and (**b**) depth error and (**c**) and (**d**) width error.

Figure 15 illustrates the surface plots of dimensional accuracy for the significant RUM parameters. In general, the percentage of depth error and width error of the machined microchannels does not exceed 11% under any combination of the RUM input parameters employed in this study. However, minimum values of DE and WE (2–3%) are found at lower levels of depth of cut and feed rate and higher levels of the ultrasonic frequency and amplitude, as can be seen in Figure 15. This implies that any combination of the RUM parameters that reduces the cutting forces such as lower depth of cut and higher amplitudes (see Figure 15) will result in lower DE and WE.

### 3.4. Tool Wear

**Figure 16:**
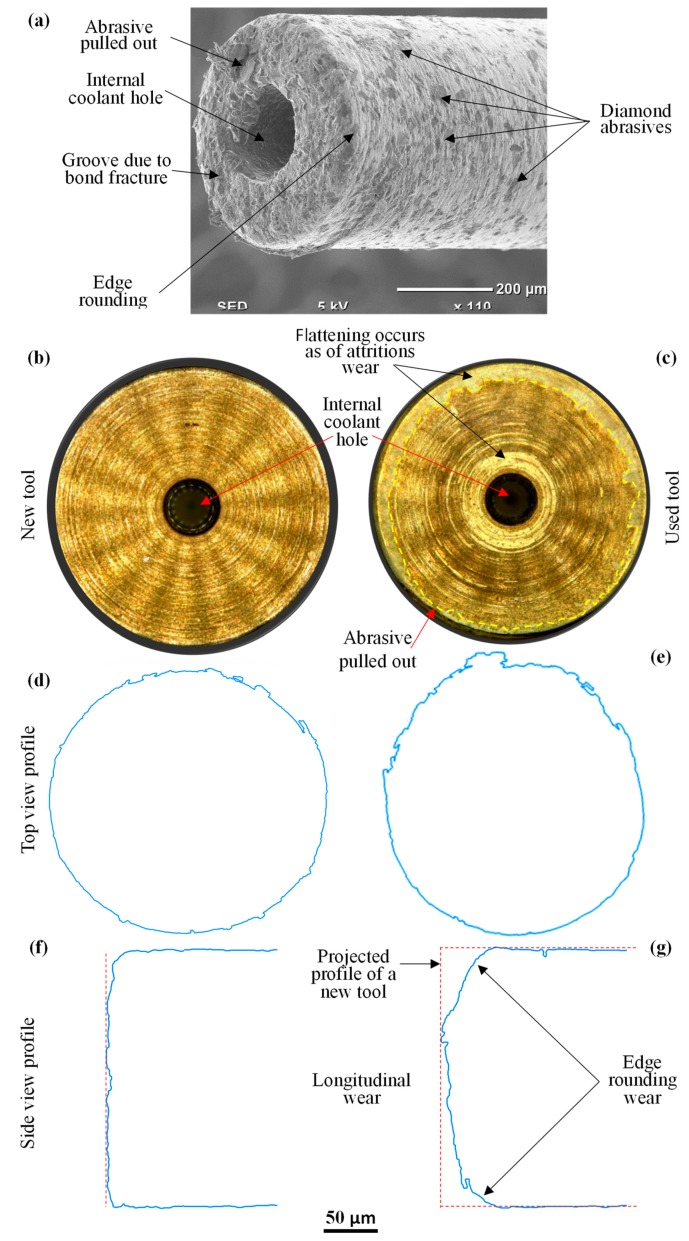
Tool wear: (**a**) a 3D view of the used RUM tool; (**b**) end cutting face of the new RUM tool; (**c**) end cutting face of the used RUM tool; (**d**) end face profile of the new tool; (**e**) end face profile of the used tool; (**f**) side face profile of the new tool; and (**g**) side face profile of the used tool.

Each µ-RUM tool was used to machine 11 microchannels. The comparison of the new RUM tool and the tool after machining 11 channels is shown in Figure 16. In the SEM micrograph of the used tool (see Figure 16a) various wear mechanisms including the attritious wear, diamond pulled out, bond fracture, and edge rounding are observed; these tool wear mechanisms have also been reported in [22,37]. A comparison of the end faces of the used and new tools is presented in Figure 16b,c. The used tools exhibited high tendency of tool wear around the edges and near the center of the tool [38]. The profiles of end faces and the side faces of the new and used tools are compared in Figure 16d,e and Figure 16f,g, respectively. In the case of the end face of the used tool, the profile of the tool (the active contact edge) deviated from its true circular form due to tool wear. Regarding the profile of the side face, the used tool shows an edge rounding effect (see Figure 16e) due to several active tool wear mechanisms, which can adversely affect the geometry of the machined microchannels. The effect of the tool edge rounding can be seen in the microchannel geometry in Figure 13a, where at the bottom of the channel’s wall, round instead of sharp corners are produced. Furthermore, the length of the used tool slightly decreases in comparison to the new tool due to the longitudinal wear (see Figure 16g) [20]. However, the tool length wear did not affect the microchannel geometry, since after machining each microchannel the tool length was calibrated by using the DMG laser tool length compensation system. 

### 3.5. Optimization Results

**Figure 17:**
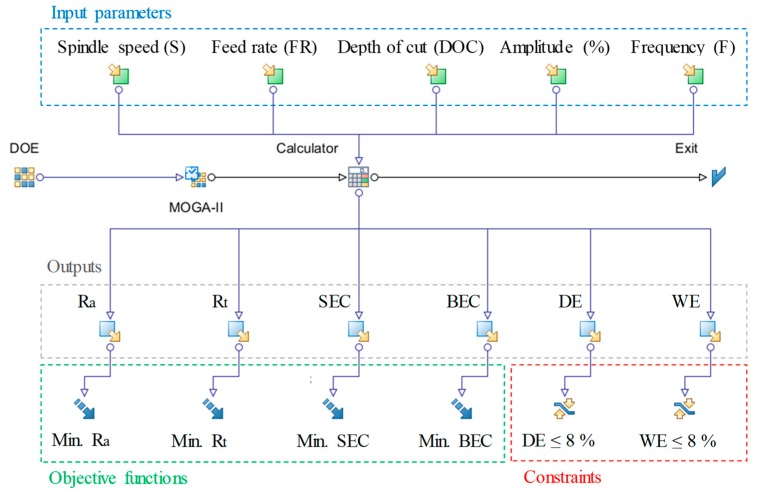
Process flow for the optimization problem.

**Figure 18:**
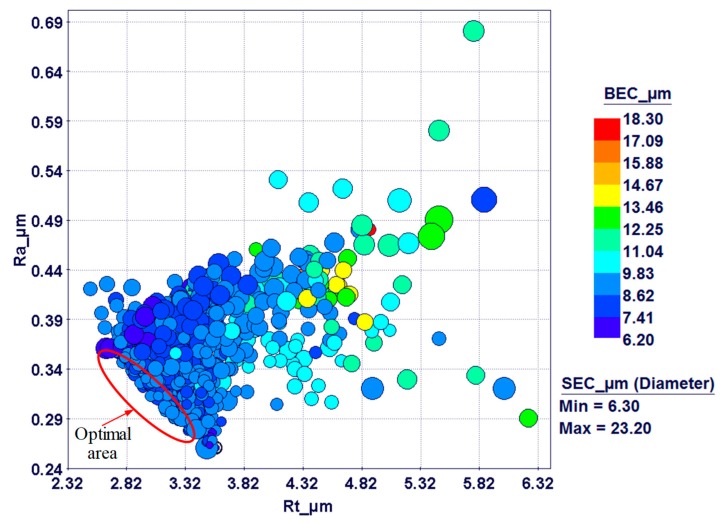
A 4D bubble chart showing the design points obtained with outputs of Ra, Rt, SEC, and BEC.

**Figure 19:**
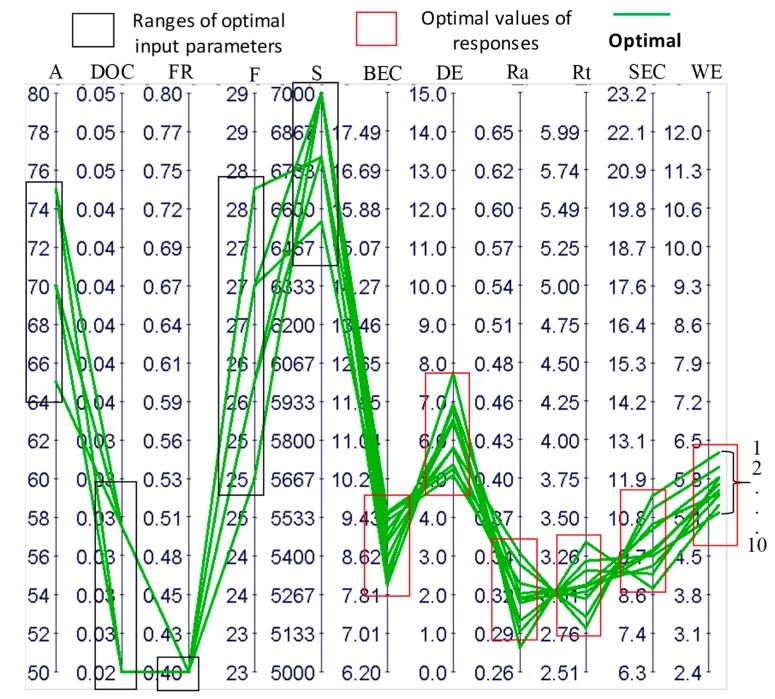
A parallel coordinate chart for the analysis of RUM parameters for optimal points.

The current work, besides studying the effect of the RUM parameters on the outputs, also attempts to optimize the RUM parameters. A multi-objective genetic algorithm was utilized to minimize the surface roughness (Ra and Rt), side exit chipping, and bed exit chipping of the milled microchannels. Response surfaces based on radial basis functions were used in the optimization algorithm and applied to predict responses for a specific design point. A percentage of the depth error and width error greater than 8% was considered an unfeasible solution in the optimization model. The objective functions and the constraints used in the optimization model are presented in Table 4. Figure 17 illustrates the process flow for the optimization problem developed by utilizing the Mode Frontier® program. The optimization problem was modelled based on the multi-objective genetic algorithm. The selected parameters of the MOGA are presented in Table 5. The total number of design points is equal to the product of the number of generations and the number of design points in the CCD table (see Table 3). Detailed information concerning the MOGA can be seen in [39]. A total of 3200 generations were modelled using the MOGA. Figure 18 and Figure 19 show the design points obtained using the bubble charts. The real design points related to the actual experimental runs and the virtual runs are predicted by the response surfaces.

The four responses at a time obtained using a 4D bubble chart are shown in Figure 18. The diameter of the bubbles represents the side exit chipping while the colors represent the bed exit chipping. The effect of Ra and Rt on SEC and BEC is clearly established in the 4D chart, as the high Ra and Rt area corresponds to higher BEC and SEC. These design points are distinguished by low cutting speed and frequency and high feed rate, depth of cut, and amplitude. On the other side, the bottom left area of Figure 18 represents design points with low Ra and Rt. These are associated with low feed rate and depth of cut and amplitude, high cutting speed, and moderate to high levels of frequencies. In general, the variation of SEC and BEC is not high, and most of the values are located between 6 to 10 µm. This can be observed by the difference in the color and size of the bubbles in Figure 18.

Optimal results were found as having moderate to high levels of amplitudes and frequencies (64–75% and 25 kHz–28 kHz, respectively), low levels of depth of cut (0.25–0.31 μm), a low level of feed rate (0.4 mm/min), and high levels of cutting speed (6500 rpm–7000 rpm). The optimal design points are shown in Figure 19 and listed in Table 6. 

## 4. Conclusions

In this study, an experimental analysis was executed to examine the influence of five major RUM input parameters on surface roughness, edge chipping, and dimensional accuracy on microchannels milled on alumina (Al_2_O_3_) bioceramic. Multi-objective optimization was also carried out using a MOGA tool. The following conclusions can be drawn from the present study.

Among all the selected input parameters, the feed rate and spindle speed were the most influential parameters affecting the surface roughness (Ra) of the machined microchannels. Lower Ra was achieved at higher spindle speed and lower feed rates due to increases in diamond abrasive cutting action and reductions in brittle fracture.

- Vibration frequency significantly affects crack density and crack propagation and hence affects the depth of micro-pits generated in the machined surface, consequently influencing the Rt;
- In general, a smoothed surface morphology with more plastic removal, less brittle fracture, and low exit edge chipping can be achieved by employing a high spindle speed (7000 rpm) and a lower feed rate (0.4 mm/min) and depth of cut (0.025 mm), and medium to high levels of vibration amplitude (65–75%) and frequency (25 kHz–28 kHz);
- RUM can be applied to mill precise microchannels in Al_2_O_3_ bioceramic. The dimensional accuracy in terms of the depth error and width error is found to be less than 11% at any combination of the selected RUM parameters;
- A multi-objective optimization method was successfully accomplished in order to minimize the surface roughness (Ra and Rt) and the exit edge chipping (SEC and BEC) while keeping the dimensional errors (DE and WE) less than 8%;
- The current study provides the optimal parametric combination of RUM parameters to obtain microchannels in Al_2_O_3_ with high surface quality and accuracy. The optimal RUM parameters are a cutting speed of 7000 rpm, a feed rate of 0.4 mm/min, a depth of cut of 0.025 mm, a frequency of 27 kHz, and an amplitude of 75%, yielding the key responses as Ra = 0.27 μm, Rt = 2.7 μm, SEC = 8.7 μm, BEC = 8 μm, DE = 5%, and WE = 5.2%.

## Figures and Tables

**Table 1:** Mechanical and thermal properties of alumina materials [26].

Property	Value (unit)
Flexural strength (20 °C)	630 (MPa)
Compressive strength	4000 (MPa)
Tensile strength	650 (MPa)
Bulk density	3.96 (g/cm^3^)
Poisson’s ratio	0.23
Vickers hardness (HV 0.5)	2000
Fracture toughness	4 (MPa·m^1/2^)
Thermal conductivity 20°C	30 (W/mK)
Melting point	2270 (°C)

**Table 2:** RUM parameters and their levels.

**Parameter (unit)/Levels**	1	2	3
**Spindle Speed (rpm)**	5000	6000	7000
**Feed rate (mm/min)**	0.4	0.6	0.8
**Depth of cut (mm)**	0.025	0.0375	0.05
**Vibration amplitude % (** **μm)**	50 (5)	65 (27.5)	80 (50)
**Vibration frequency (kHz)**	23	26	29

**Table 3:** Experimental results.

Exp. #	Input Parameters					Responses					
	Speed (rpm)	Feed rate (mm/min)	Depth of cut (mm)	Frequency (kHz)	Amplitude (%)	SEC (µm)	BEC (µm)	Ra (µm)	Rt (µm)	DE (%)	WE (%)
1	5000	0.4	0.025	29	50	17.6	6.2	0.36	2.65	5.5	9.0
2	7000	0.8	0.05	23	50	19.2	9.3	0.32	6.03	11.2	9.0
3	6000	0.6	0.05	26	65	14.6	10.6	0.42	3.96	6	10.8
4	7000	0.4	0.05	23	80	9.6	10.3	0.29	3.33	10	9.4
5	6000	0.4	0.0375	26	65	12.5	9.5	0.42	2.51	8	9.0
6	6000	0.6	0.0375	26	65	16.7	10.2	0.39	3.19	11	11.6
7	5000	0.4	0.05	23	50	17.6	11.6	0.68	5.77	13	6.6
8	5000	0.8	0.025	23	50	16.9	9.2	0.48	4.81	7.5	7.8
9	5000	0.8	0.05	23	80	23.2	13.5	0.49	5.48	11.9	11.7
10	7000	0.8	0.025	23	80	19.2	9.2	0.32	4.91	9	4.8
11	7000	0.4	0.025	29	80	6.3	6.6	0.26	2.58	12	5.6
12	6000	0.6	0.0375	26	65	15.9	12.3	0.40	4.11	8	4
13	6000	0.6	0.0375	26	80	14.7	11.2	0.35	4.33	4	9
14	7000	0.8	0.025	29	50	11.6	8.2	0.39	4.76	15	7
15	5000	0.6	0.0375	26	65	21.3	8.6	0.51	5.86	8	7.8
16	5000	0.4	0.05	29	80	19.6	12.7	0.43	4.06	2.5	2.4
17	6000	0.6	0.0375	29	65	19.1	7.1	0.42	3.38	3.5	4.4
18	7000	0.8	0.05	29	80	16.3	13.3	0.29	6.24	9	2.8
19	6000	0.8	0.0375	26	65	17.2	17.5	0.43	4.29	5.5	7.4
20	6000	0.6	0.0375	26	65	14.7	11.7	0.41	3.46	11.5	5.8
21	6000	0.6	0.0375	26	65	12.3	12.6	0.23	3.5	8	3.4
22	6000	0.6	0.0375	26	65	9.7	11.3	0.44	4.22	9	2.0
23	5000	0.8	0.025	29	80	11.5	18.3	0.48	4.89	0.5	11.4
24	7000	0.4	0.025	23	50	18.9	9.9	0.26	3.5	0	3.8
25	6000	0.6	0.025	26	65	17.3	13.7	0.41	4.29	3	5.8
26	6000	0.6	0.0375	26	65	19.4	12.4	0.53	4.3	3	8.4
27	6000	0.6	0.0375	26	50	12.6	13.6	0.46	3.92	9	6.4
28	6000	0.6	0.0375	23	65	16.3	10.3	0.53	4.11	11	9.2
29	7000	0.4	0.05	29	50	12.3	9.2	0.37	5.48	2.5	5.4
30	5000	0.4	0.025	23	80	9.4	11.5	0.33	5.24	0.5	4.8
31	7000	0.6	0.0375	26	65	9.3	12.3	0.42	3.36	2.5	4.6
32	5000	0.8	0.05	29	50	17.9	12.6	0.58	5.48	3.5	7.4

**Table 4:** Optimization model used for the study.

**Objective Functions**	1. Minimize Ra 2. Minimize Rt 3. Minimize SEC 4. Minimize BEC
**Constraints**	1. Depth error ≤ 8% 2. Width error ≤ 8%

**Table 5:** Multi-objective genetic algorithm (MOGA) parameters used.

Parameter	Value
Number of generations	100
Probability of direction cross-over	0.5
Probability of selection	0.05
Probability of mutation	0.1
DNA string mutation ratio	0.05
Random generator seed	1

**Table 6:** Optimal points for all responses.

Point #	Inputs					Outputs					
	S	FR	DOC	F	A	SEC	BEC	Ra	Rt	DE	WE
1	7000	0.4	0.031	25	70	10.45	9.52	0.313	3.067	5.352	5.854
2	7000	0.4	0.031	26	65	10.60	9.35	0.320	2.978	5.092	5.560
3	6777.7	0.4	0.031	26	65	11.05	9.47	0.337	2.865	5.247	6.029
4	7000	0.4	0.031	26	75	9.15	8.98	0.296	3.161	6.501	5.838
5	7000	0.4	0.031	26	70	9.78	9.15	0.312	3.024	5.784	5.725
6	7000	0.4	0.031	27	70	9.36	8.58	0.309	3.064	6.451	5.533
7	6555.5	0.4	0.031	27	65	11.43	8.90	0.346	2.789	5.807	6.284
8	7000	0.4	0.025	27	75	8.74	8.13	0.277	3.343	7.740	5.361
9	7000	0.4	0.025	27	70	9.67	8.30	0.289	3.220	6.818	5.223
10	6777.7	0.4	0.031	28	70	9.86	8.01	0.316	3.058	6.926	5.615
